# Pulmonary Tuberculosis Presenting As Septic Shock in an Immunocompetent Patient: Revisiting an Old Disease With New Perspectives

**DOI:** 10.7759/cureus.37362

**Published:** 2023-04-10

**Authors:** Manika Malik, Naman Lodha, Durga S Meena, Binit Sureka

**Affiliations:** 1 Internal Medicine, All India Institute of Medical Sciences, Jodhpur, IND; 2 Diagnostic and Interventional Radiology, All India Institute of Medical Sciences, Jodhpur, IND

**Keywords:** pulmonary, immunocompetent, septic shock, sepsis, tuberculosis

## Abstract

Septic shock due to *Mycobacterium tuberculosis (M. tuberculosis)* in immunocompromised patients (particularly HIV) is a well-recognized clinical entity. However, tubercular sepsis in the immunocompetent is still underdiagnosed and under-discussed. Moreover, sepsis is usually associated with gram-negative and other gram-positive microorganisms that can cause similar pulmonary and disseminated disease and can further convolute the diagnosis. We herein discuss a case of an elderly female who presented with acute onset fever, cough, and altered talk from the last seven days. Her initial clinical and laboratory examination revealed features of lower respiratory tract infection with septic shock. She was started on broad-spectrum antibiotics based on severe community-acquired pneumonia management guidelines. Her blood and urine cultures were sterile. She did not respond to initial antibiotics. Furthermore, sputum production was not possible, which compelled us for gastric aspirate analysis, which came positive for cartridge-based nucleic acid amplification test (CBNAAT). In repeated blood cultures, *M. tuberculosis* was also isolated. She was started on antitubercular treatment; on the 12th day of antitubercular treatment, she developed acute respiratory distress and eventually succumbed to her illness on the 19th day of hospitalization. We highlighted the importance of early diagnosis and prompt antitubercular therapy in tubercular septic shock. We also discuss the possibility of tubercular-immune reconstitution inflammatory syndrome (IRIS) in such patients, which could be a contributing factor to mortality.

## Introduction

Sepsis is a life-threatening clinical syndrome characterized by dysregulated host immune response to infection [1]. Sepsis is still the predominant cause of death (nearly 20% of global deaths), particularly in the subgroup related to septic shock [2]. Disseminated tuberculosis has a myriad of clinical manifestations. However, sepsis or septic shock in tuberculosis is uncommon, especially in immunocompetent individuals. In literature, most cases of tubercular sepsis are described in association with an immunocompromised state [3], especially with HIV. Tubercular sepsis was attributed to less than 1% of all cases of bacterial sepsis, with the majority of cases belonging to the immunodeficiency state [4]. We herein describe a case of disseminated tuberculosis presented as a septic shock in an elderly immunocompetent female. Due to high mortality, a high index of suspicion is warranted for early diagnosis and better outcomes.

## Case presentation

A 70-year-old female patient from Western Rajasthan with no known co-morbidities presented to the emergency room with a history of low-grade undocumented fever, dry cough, and altered talk from the last seven days. The presenting complaints were acute onset and rapidly progressive. The patient became bedridden in the last two days before her current hospitalization. Her past history was unremarkable. There was no history of illicit drug use. On examination, the patient had altered talk; she was also febrile, pale, and icteric. Further examination showed a pulse rate of 112 beats/min, respiratory rate of 16/min, Spo2 of 90% on room air, and blood pressure of 80/54 mm hg. Neurological examination revealed a Glasgow Coma Scale of 13 (E4V3M6), planter reflex were flexor; in addition, there were no meningeal signs. Rest systematic examination revealed bilateral basal diffused crepitation and palpable spleen (2 cm below right costal margin). On laboratory investigations, the patient had elevated total leukocyte counts (TLC), raised inflammatory and sepsis markers, low platelet count, deranged liver and renal function tests, coagulopathy, and low calcium levels (Table 1).

**Table 1:** Biochemical and haematological investigations AST -aspartate aminotransferase, ALT - alanine aminotransferase, ALP - alkaline phosphatase, CRP - C-reactive protein, LDH - lactate dehydrogenase, INR - international normalized ratio, HCV - hepatitis C virus

Laboratory parameters		Patient values	Reference values
Hemoglobin		9.9 g/dl	12-15 g/dl
Total leukocyte counts		36.17 x 10^3 ^/uL	(4-11) ×10^3^/mL
Platelets		90 x 10^3 ^/uL	150-400 x 10^3 ^/uL
Liver function tests	AST	159 IU/L	<35 IU/L
	ALT	126 IU/L	<35 IU/L
	ALP	585 IU/L	40-120 IU/L
	Total bilirubin	13.08 mg/dl	0.3-1.2 IU/L
	Direct bilirubin	7.85 mg/dl	<0.2 IU/L
	Indirect bilirubin	5.23 mg/dl	0.2-1.2 IU/L
	Total protein	3.86 gm/dl	6.6-8.3 gm/dl
	Albumin	1.71 gm/dl	3.5-5.2 gm/dl
	Globulin	2.15 gm/dl	2-3.5 gm/dl
Renal function tests	Serum urea	194 mg/dl	17-43 mg/dl
	Serum creatinine	2.53 mg/dl	0.66-1.09 mg/dl
Serum electrolytes	Sodium	133 Meq/L	135-145 Meq/L
	Potassium	4.35 Meq/L	3.5-5.0 Meq/L
	Calcium	7.24 mg/dl	8-10.5 mg/dl
Inflammatory markers	CRP	85.86 mg/dl	<1 mg/dl
	Procalcitonin	4.11 ng/ml	<0.04 ng/ml
Viral markers	HIV	negative	
	HBsAg	negative	
	Anti-HCV	negative	
LDH		857 IU/L	105-333 IU/L
D-dimer		12 ug/ml	<0.5 ug/ml
INR		1.3	<1.1
CD4 counts		643 cells/mm^3^	500-1500 cells/mm^3^
Blood film morphology	RBC - normocytic normochromic to microcytic hypochromic RBCs with few macro-ovalocytes; WBC - neutrophilic leukocytosis, No hemoparasites seen		

**Figure 1:**
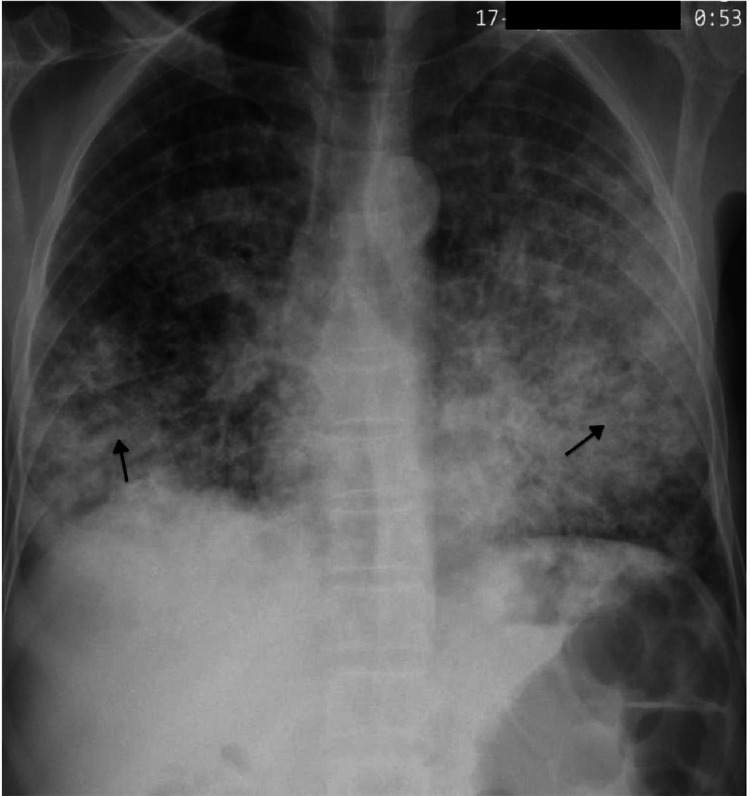
X-ray chest showing bilateral diffuse heterogeneous opacities (black arrows)

The provisional diagnosis of lower respiratory tract infection (LRTI) with sepsis and multiorgan organ dysfunction (MODS) was kept. Initially, antibiotics were selected based on the probable etiology of community-acquired pneumonia (CAP) (e.g., *Streptococcus pneumoniae*, *Haemophilus influenzae*, and other atypical organisms). Subsequently, the patient was started on IV piperacillin-tazobactam and azithromycin. All initial blood cultures/urine cultures were sterile. X-ray showed bilateral diffuse heterogeneous opacities (Figure 1).

**Figure 2:**
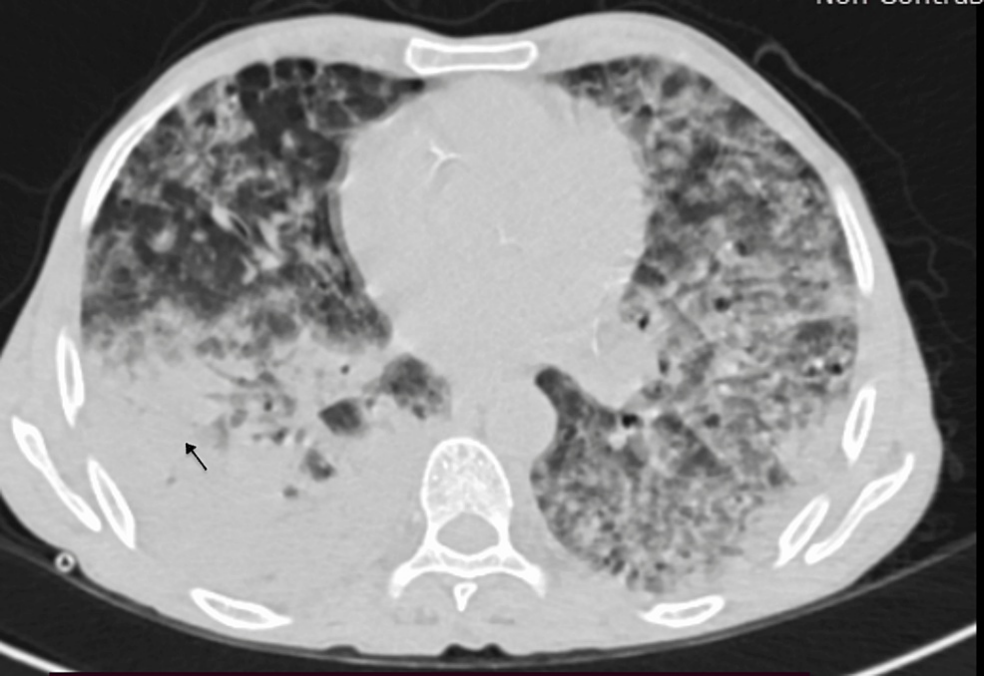
CT thorax showing large confluent areas of consolidation (black arrow), seen involving both the lungs (right lower lobe, left upper and lower lobes) with areas of ground glass opacities in rest of the bilateral lung parenchyma

Computed tomography imaging of the thorax showed large confluent areas of consolidation seen involving both the lungs (right lower lobe, left upper and lower lobes) with areas of ground glass opacities in the rest of the bilateral lung parenchyma (Figure 2).

Multiple enlarged lymph nodes were also seen in the mediastinum, involving paratracheal and subcarinal (largest ~ 17 mm SAD, subcarinal). Abdominal CT imaging demonstrated splenomegaly with ill-defined hypodense areas in the liver and spleen (likely infarcts). CT abdomen also showed focal hepatic necrosis in segment VIII of the liver and mild ascites with diffuse mesenteric and subcutaneous fat stranding. She was put on vasopressor support (noradrenaline infusion) due to her non-responsiveness with fluid resuscitation. On day five of hospitalization, she worsened with no improvement in clinical and laboratory indices except blood pressure. Piperacillin-tazobactam was substituted with meropenem and linezolid. COVID-19 was ruled out by performing COVID reverse transcription polymerase chain reaction (RT-PCR) test which was negative. The possibility of pneumocystis jirovecii pneumonia was unlikely (negative beta-D-glucan, and normal lactate dehydrogenase levels), however bronchoscopy could have been more useful in this case. We also ruled out the cardiac causes (2D echo was unremarkable). After repeated failed trials to induce sputum, bronchoscopy was considered to further explore the underlying etiology. However, due to the hemodynamic instability of the patient, her relatives did not provide consent for bronchoscopy. Finally, we decided to send the gastric aspirate for a cartridge-based nucleic acid amplification test (CBNAAT), which was reported positive on day seven of admission. The patient was started on modified antitubercular therapy (ATT) because of deranged liver function. On day 14 of hospitalization, liver enzymes returned to normal, and first-line ATT was introduced according to British Thoracic Society guidelines. The patient showed improvement both clinically (improvement in sensorium and respiratory symptoms), and in terms of laboratory parameters. In follow-up lab parameters, improvement was observed in hemoglobin (10.4 g/dl), platelet counts (156 x 10^3^ /uL), C-reactive protein (13 mg/dl), and procalcitonin (0.8 ng/ml). However, TLC was persistently elevated, showing neutrophilic leukocytosis (N92%, L7%). On further evaluation, this was attributed to leukemoid reaction. She did not have any overt immunodeficiency state; her CD4 counts were also normal (Table 1). After showing initial improvement, the patient again developed progressive shortness of breath with severe hypoxemia (spo2 85%). On day 19 of admission, she was put on mechanical ventilation owing to worsening respiratory symptoms. Her arterial blood gas (ABG) analysis showed type-1 respiratory failure and acute respiratory distress syndrome (ARDS). The patient was reevaluated for sudden deterioration, a repeat 2D echo was normal. Her blood and tracheal aspirate cultures were sterile. She eventually succumbed to her illness on day 20 of hospitalization.

## Discussion

Septic shock is a subset of sepsis characterized by circulatory, cellular, and metabolic abnormalities associated with high mortality [1]. The mortality rate for sepsis ranges from 20 to 40%, depending on the study population [5,6]. Tuberculosis is a widely recognized entity, especially in developed countries; however, little is known about tubercular septic shock. There is an ambiguity regarding its incidence, risk factors, clinical presentation, and outcomes. A retrospective study by Kethireddy et al. first describes the epidemiology, risk factors, and predictors of survival in tubercular septic shock [4]. In their report, tubercular septic shock was predominantly associated with immunocompromised patients. Similarly, a meta-analysis by Barr et al. reported the high incidence of tubercular bloodstream infections in HIV patients [3]. They found that the estimated prevalence of tubercular bloodstream infections was 38% in HIV patients [3]. However, there was a large heterogenicity in data, with a lot of confounding factors like the study population. Some of the studies included in the meta-analysis reported a prevalence of around 1-2% [7]. Tubercular sepsis shock in immunocompetent patients is rare, with few reports published so far [8-12]. Notwithstanding, our patient presented with a short history of fever followed by altered sensorium and septic shock, which mimicked the more common gram-negative sepsis and made the diagnosis difficult. 

In this case, the patient had a short history of fever, cough, and shortness of breath (seven days) and presented with septic encephalopathy. In such settings, the diagnosis of tuberculosis is difficult to establish. Community-acquired pneumonia (bacterial or viral) is the usual etiology in this clinical scenario. For laboratory confirmation, sputum acid-fast bacilli (AFB) or CBNAAT positivity is required, which was negative in many cases, resulting in further delay in diagnosis and erroneous treatment by antimicrobials. All these factors contribute to the high mortality in tubercular septic shock. In our case, the patient was initially diagnosed with community-acquired severe pneumonia and received IV meropenem. The initial blood cultures and sputum analysis were negative. Partial response (maybe due to the antitubercular effect of meropenem) and persistent leukocytosis prompt us to explore the possibility of tuberculosis. Gastric aspirate analysis for CBNAAT was vital in our case to establish the diagnosis. In the pediatric population, a gastric aspirate is frequently used for diagnosis. Now evidence is also emerging in favor of its use in adult (especially in elderly) patients [13,14]. According to a recent report, molecular analysis of gastric aspirate can yield the diagnosis of tuberculosis in 48% of the cases [14]. Elderly patients who remain unconscious usually swallow their sputum, and gastric aspirate is one of the non-invasive methods which can evade the use of bronchoalveolar lavage in such patients. 

Based on the available literature, the mean TLC of tubercular septic shock usually remains within the normal range and is significantly lower than bacterial sepsis [4]. However, in our case, it was around 37 x 103 /uL on admission, which again favors bacterial sepsis. Furthermore, serum procalcitonin (PCT) level was also significantly raised (4.11 ng/ml, normal <0.04). The various reports highlighted the role of serum PCT levels in separating the pneumonia etiology (high PCT levels in CAP compared to tubercular) [15,16]. All these factors (in addition to negative sputum examination) made the early diagnosis of tubercular sepsis difficult, like in our case, and delayed the initiation of antitubercular therapy. Tubercular septic shock carries high mortality compared to other bacterial septic shock (79.2% vs. 49.7%) [4]. Kethireddy et al. highlighted the role of early initiation of ATT in tubercular septic shock to reduce mortality [4]. According to their report, the persistence of septic shock and delayed initiation of ATT (after 24 hours) were significant predictors of mortality [4]. In our case, though septic shock was reverted after vasopressor support, ATT was started on day seven after the molecular confirmation of disseminated tuberculosis. Another significant predictor of mortality is the isolation of *Mycobacterium tuberculosis* (MTB) from blood, which was reported by Kethireddy et al., all patients with MTB bacteremia died in their study. In this case, MTB was subsequently isolated from blood on day eight of hospitalization. Another intriguing issue is the clinical response soon after the initiation of ATT. The leading cause of death in pulmonary tubercular sepsis is the persistence of septic shock and multi-organ dysfunction syndrome (MODS) or respiratory failure. Our patients had rapid clinical deterioration after ATT initiation, owing to the paradoxical immune reconstitution inflammatory syndrome (IRIS). Though, a bronchoscopy could not be performed, which may have helped in further delineating the cause. These patients usually have a significant bacillary load which could be the instigating factor for TB-IRIS. Rapid killing of mycobacteria and release of MTB antigen produce exaggerated inflammatory responses resulting in TB-IRIS. Literature is scanty regarding the management of TB-IRIS in non-HIV patients. Whether corticosteroid use would have been helpful in this scenario is a matter of discussion. 

## Conclusions

Septic shock is an underdiagnosed condition in pulmonary tuberculosis, especially in immunocompetent patients, that can easily be overlooked due to more common bacterial etiologies. A high index of suspicion for prompt diagnosis and early initiation of ATT is warranted to curb the high mortality. The utility of gastric aspirate analysis should be explored in cases with inconclusive sputum examination. Further studies will be needed to understand the role of corticosteroids in TB-IRIS.
